# Fabrication of photothermally active poly(vinyl alcohol) films with gold nanostars for antibacterial applications

**DOI:** 10.3762/bjnano.9.193

**Published:** 2018-07-23

**Authors:** Mykola Borzenkov, Maria Moros, Claudia Tortiglione, Serena Bertoldi, Nicola Contessi, Silvia Faré, Angelo Taglietti, Agnese D’Agostino, Piersandro Pallavicini, Maddalena Collini, Giuseppe Chirico

**Affiliations:** 1 Department of Medicine and Surgery, Nanomedicine Center, University of Milano-Bicocca, Piazza dell’Ateneo Nuovo, 20126, Milan, Italy; 2 Institute of Applied Sciences and Intelligent Systems, National Research Council of Italy, Via Campi-Flegrei 34, 80078, Pozzuoli, Italy; 3 Politecnico di Milano, Department of Chemistry, Materials and Chemical Engineering “Giulio Natta”, Piazza Leonardo da Vinci 32, 20133, Milan, Italy; 4 Local Unit Politecnico di Milano, INSTM, Consorzio Nazionale di Scienza e Tecnologia dei Materiali, Milan, Italy; 5 Department of Chemistry and Centre for Health Technology, University of Pavia, Viale Taramelli 12, 27100, Pavia, Italy,; 6 Department of Physics “G. Occhialini”, University of Milano-Bicocca, Piazza dell’AteneoNuovo, 20126, Milan, Italy

**Keywords:** antibacterial properties, gold nanostars, photothermal effect, poly(vinyl alcohol) films

## Abstract

The unique photothermal properties of non-spherical gold nanoparticles under near-infrared (NIR) irradiation find broad application in nanotechnology and nanomedicine. The combination of their plasmonic features with widely used biocompatible poly(vinyl alcohol) (PVA) films can lead to novel hybrid polymeric materials with tunable photothermal properties and a wide range of applications. In this study, thin PVA films containing highly photothermally efficient gold nanostars (GNSs) were fabricated and their properties were studied. The resulting films displayed good mechanical properties and a pronounced photothermal effect under NIR irradiation. The local photothermal effect triggered by NIR irradiation of the PVA-GNS films is highly efficient at killing bacteria, therefore providing an opportunity to develop new types of protective antibacterial films and coatings.

## Introduction

The photothermal properties of non-spherical gold nanoparticles possessing localized surface plasmon resonance (LSPR) located in NIR range has already been explored in numerous studies to eradicate cancer cells, controlled drug delivery and enhancement of cell growth [1–5]. Among the many types of such nanoparticles, gold nanostars (GNSs) (having a well-tunable LSPR position in the biotransparent NIR window (750–1300 nm) and ease of surface decoration) have received particular attention [6–7]. Previously, it was shown that these GNSs are highly photothermally active under NIR irradiation in aqueous solution in the form of monolayers grafted on dry glass surfaces and when inkjet-printed on flexible substrates, demonstrating the ability to reach higher temperatures (Δ*T* > 40 °C) in the latter case [3,8–10]. Poly(vinyl alcohol) (PVA) polymer is also an attractive material for GNS-based film fabrication, as it displays a range of useful properties, namely, low toxicity, biocompatibility, hydrophilicity, chemical stability, and excellent film-forming properties [11–12]. All these properties encouraged extensive investigations focused on fabrication of a diversity of PVA-based materials for various industrial and medical applications, including food packaging materials, wound dressing substrates, etc. [13–16]. In addition, numerous studies have reported the preparation and the antibacterial efficacy of PVA films containing plant extracts, silver nanoparticles or zinc oxide nanoparticles [17–22]. However, when bacteria start to form biofilms they become resistant and conventional antibiotics do not eradicate biofilms from the surfaces where they formed [8]. Silver nanoparticles prevent biofilm formation but with a controversial results – Ag nanoparticles are effective towards Gram-negative bacterial strains but much less so towards Gram-positive [8,23]. There is a strong need for novel lightweight antibacterial materials that can coat surfaces and are capable of eradicating biofilms with remote physical activation. Even though gold nanoparticles are not intrinsically antibacterial, the thermal relaxation upon NIR light activation can be a major force of antibacterial action as it was shown for monolayers of GNSs on glass slides [8]. In this case, the efficient photothermal response of the monolayers resulted in a local hyperthermia effect that was capable of killing bacteria in *Staphylococcus aureus* biofilms [8].

Therefore, the motivation of the present work lies on the assumption that the incorporation of highly photothermally active GNSs into PVA films may lead to a new class of PVA-based antibacterial materials suitable for coating. The materials are anticipated to exhibit a strong and highly localized hyperthermal effect activated by a range of NIR wavelengths. In this study, PVA films containing five-branched gold nanostars, synthesized via a seed-growth method as previously reported [24], were fabricated. Such nanoparticles feature two intense LSPR peaks in the 600–900 and 1100–1600 nm ranges, and are able to convert laser radiation into heat, offering two photothermally active channels [24]. The fabricated films containing these nanoparticles displayed a pronounced photothermal effect upon irradiation at three laser wavelengths (λ = 730, 800 and 1064 nm). Moreover, we demonstrated that the local NIR-induced increase of temperature is sufficient to eradicate bacteria grown on the film surface.

## Results and Discussion

**Figure 1:**
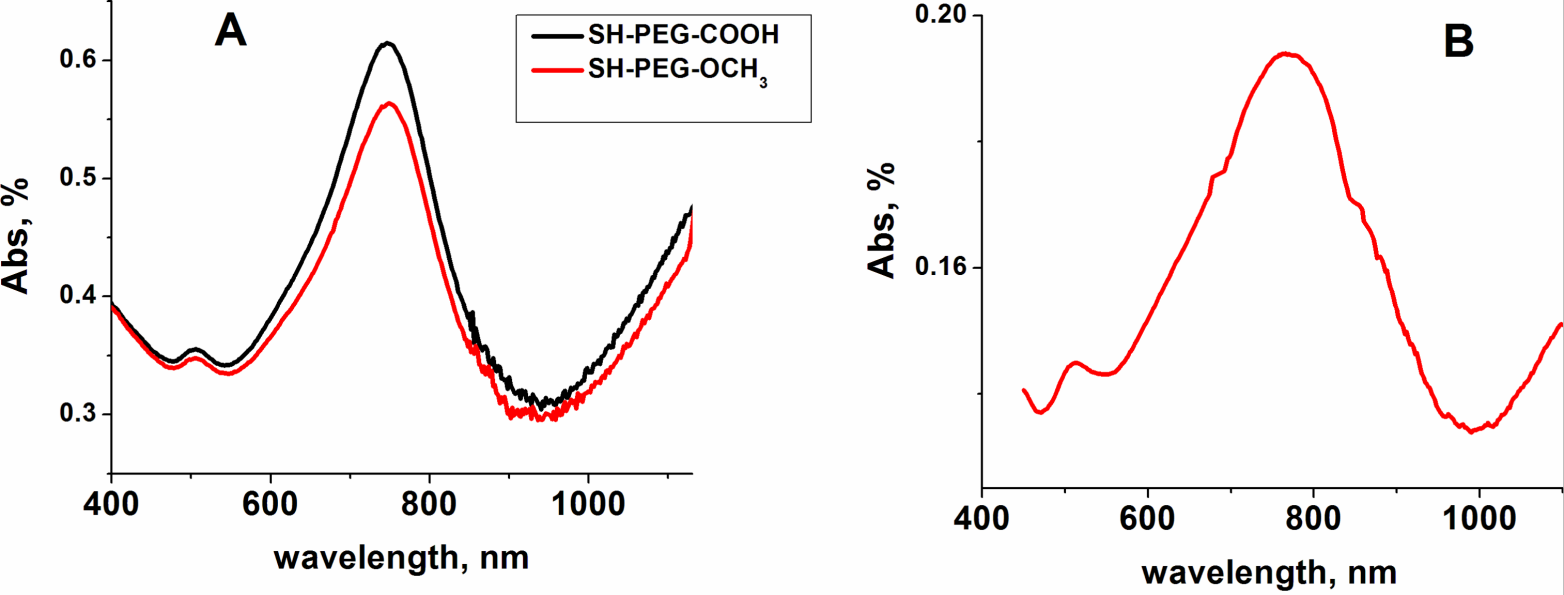
Extinction spectra of aqueous solutions of PEGylated GNSs (A) and the PVA-GNS film (B).

Crosslinked PVA films containing GNSs were prepared using a slightly modified protocol reported previously [20]. Crosslinking of the PVA matrix, together with addition of a plasticizer, poly(ethylene glycol) (PEG 200), is favorable as it can improve the stability and the mechanical properties of the films [20]. Citric acid was chosen as the crosslinking agent as, in comparison with the widely used method of crosslinking with glutaraldehyde, it is a non-toxic compound approved as a food additive, thus providing a green crosslinking route [20,25]. GNSs were synthesized via a seed-growth technique in the presence of a non-ionic surfactant, Triton X-100, according to a well-established protocol reported by our group previously [24]. An example of a typical TEM image of the resulting GNSs is provided in Supporting Information File 1 (Figure S1). Different from the GNSs obtained using the zwitterionic lauryl sulfobetaine (LSB) in the seed-growth synthesis that display NIR LSPR in the 750–1000 nm range [6], these GNSs display two intense tunable NIR LSPR peaks in the 600–900 nm and 1100–1600 nm ranges [24]. This important property enables the application of multiple NIR wavelengths to induce the photothermal effect [6,24]. Moreover, the LSPR peak located in the second biotransparent window (1000–1400 nm) is more attractive for in vivo applications due to the deeper penetration of NIR light [26]. Since GNSs are weakly stable if coated only with surfactant, and also considering the cytotoxicity of surfactants, they were coated with either SH-PEG_5000_–OCH_3_ or SH-PEG_5000_–COOH before being incorporated in the PVA matrix, as PEGylation of gold nanostars leads to enhanced stability [9–10] and complete surfactant removal [6,8].The aqueous solutions of PEGylated GNSs were prepared with 0.6 mg/mL Au concentration as determined by inductively coupled plasma optical emission spectroscopy (ICP-OES) analysis [9–10]. In addition, GNSs bearing terminal carboxylic groups may also act as additional crosslinking agents of hydroxyl groups of the PVA matrix, thus improving its mechanical resistance. Detailed information about GNS synthesis and PEGylation, PVA film preparation and all measurements is provided in the Experimental section. Using the solvent casting method we obtained uniform films with a thickness of 90 ± 15 μm. The extinction spectra of PEGylated GNS solutions and the PVA-GNS film are shown in Figure 1.

The incorporation of GNSs decreased the transparency of the PVA films (which are almost 100% transparent when not loaded with GNSs) and are observed to be semi-transparent with a light blue color. The images of fabricated films without and with GNSs are shown in Supporting Information File 1 (Figure S2).

**Figure 2:**
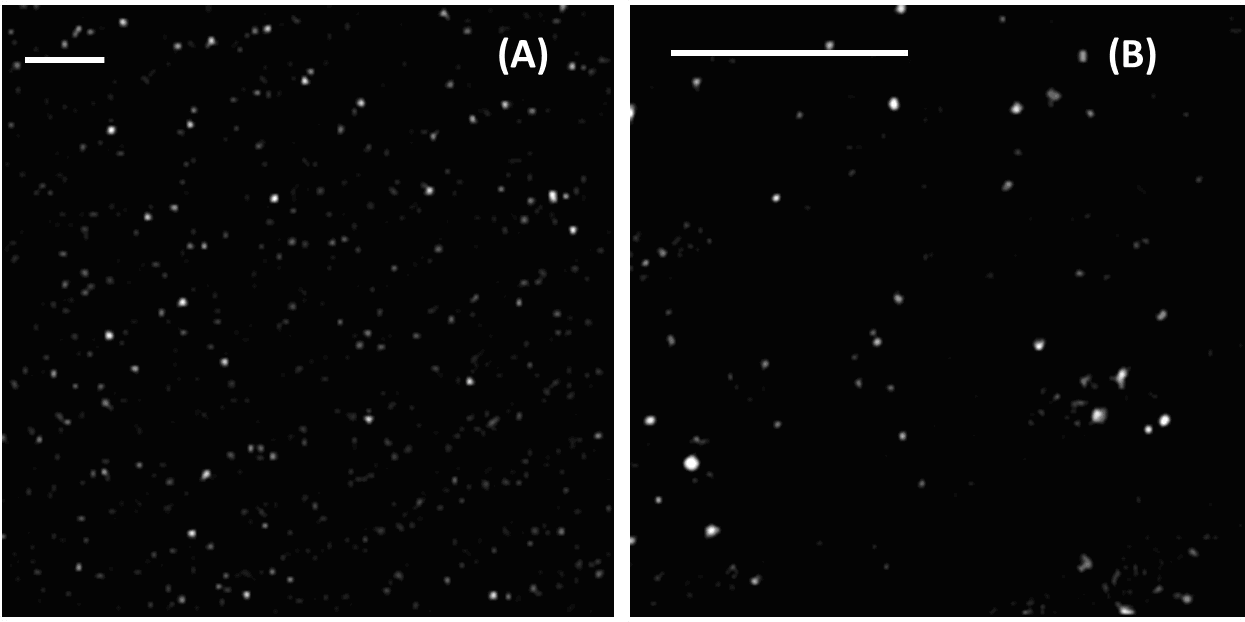
The confocal reflection images of PVA films containing gold nanostars. Field of view = 77.2 × 77.2 μm (panel A); field of view = 25.8 × 25.8 μm (panel B). Z-resolution ≈ 1.2 μm. The white scale bars indicate 10 μm.

We studied the distribution of the GNSs within the PVA films by means of reflection confocal microscopy (Figure 2). By acquiring z-stacks (20 planes, 1 μm spacing) on a 25.8 μm field of view, it was possible to estimate the density of particles in the film with 20% accuracy (see Experimental section), showing *n* = 0.015 ± 0.003 particle/μm^3^. The number of spots/layer is randomly distributed around the average value within 15% and does not depend on the distance from the film surface. This finding suggests an almost uniform distribution of the nanoparticles in the film.

Hydrophilic PVA films are known to be sensitive to water [20,27]. In order to test the potential application of the fabricated films in biological environments, a crucial prerequisite is the study of the swelling behavior and the water uptake capability [11]. With this aim, the fabricated films were immersed for 24 h in water at room temperature. At this time point, the water content of swollen films was 93 ± 0.6% with a corresponding degree of swelling of 13.3 ± 0.8%.The corresponding parameters for the PVA film without GNSs were 92.6 ± 0.6% and 13.1 ± 0.8%, respectively. This negligible difference can be explained by the fact that GNSs coated with hydrophilic PEG can slightly increase the whole hydrophilicity of the films. The weight loss of the PVA-GNS films in water as a function of time is also a relevant parameter, providing information about long-term stability of the films under prolonged use. After immersion in water for four days at room temperature the films lose 12 ± 2% of their initial weight. As previously reported, non-crosslinked PVA films under the same conditions dissolve completely [20].

**Figure 3:**
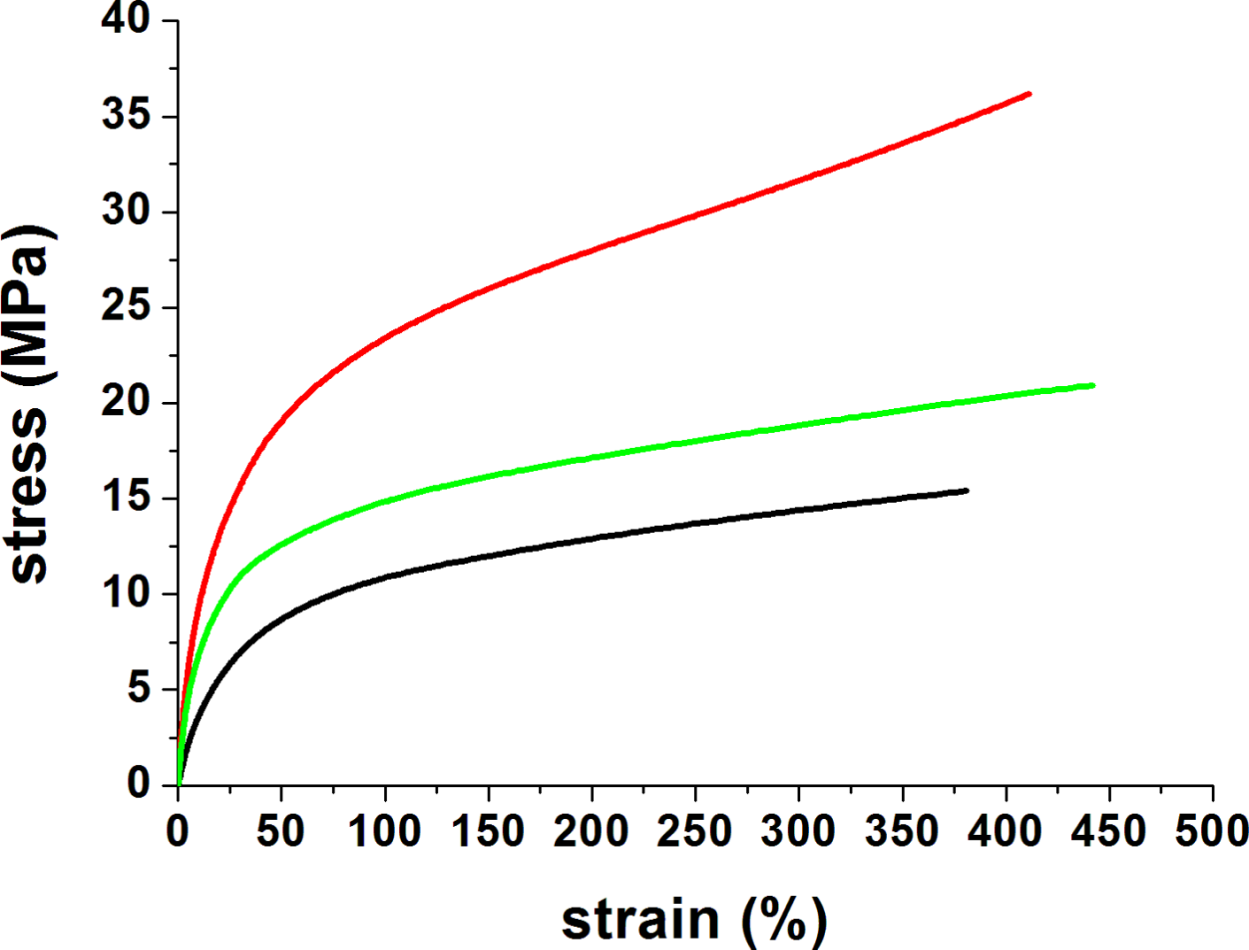
Strain-–stress curves of PVA film without gold nanostars (black); PVA film with gold nanostars coated with SH-PEG–COOH (red) and PVA film with gold nanostars coated with SH-PEG–OCH_3_ (green).

To investigate the mechanical properties, tensile studies were performed on two types of PVA-GNS films (GNSs coated with SH-PEG-OCH_3_ and SH-PEG-COOH) and on the PVA film without GNSs, as a comparison. The two different GNS coatings were selected to study the possible modification of the mechanical properties of the films induced by the presence of PEGylated GNSs, in particular when coated with PEG-bearing carboxylic groups.The representative stress–strain curves of the fabricated films are shown in Figure 3.

The detailed information about the set-up and data analysis is provided in the Experimental section**.** The mechanical parameters obtained by the stress–strain curves are reported in Table 1.

**Table 1:** Mechanical parameters obtained by tensile tests of PVA, PVA-gold nanostars (GNS) (GNSs coated with SH-PEG–COOH) and PVA-GNS (GNSs coated with SH-PEG–OCH_3_) films.

Material	Elastic modulus, *E* (MPa)	Maximum stress, σ_max_ (MPa)	Maximum strain, ε_max_ (%)

PVA film without GNSs	39 ± 3	17 ± 2	384 ± 7
PVA-GNS film (GNSs coated with SH-PEG–COOH)	112 ± 19	35 ± 2	429 ± 17
PVA-GNS film (GNSs coated with SH-PEG–OCH_3_)	95 ± 12	20.8 ± 0.9	417 ± 22

The stress–strain curves of the three types of films followed the same trend, however being characterized by different elastic modulus, maximum stress and maximum strain values. In particular, PVA films without GNSs showed the lowest stiffness value in comparison with both types of PVA-GNS films (*p* < 0.05). At the same time films prepared with GNSs coated with SH-PEG−COOH were characterized by the highest value of maximum stress when compared to films prepared with GNSs coated with SH-PEG–OCH_3_ and with bare PVA films (*p* < 0.05). All the compositions showed instead similar values of the maximum strain ≈380–420%. It is worth noting that in all cases the sample break point was not reached, as the specimens reached the limit of deformation of the testing machine.

**Figure 4:**
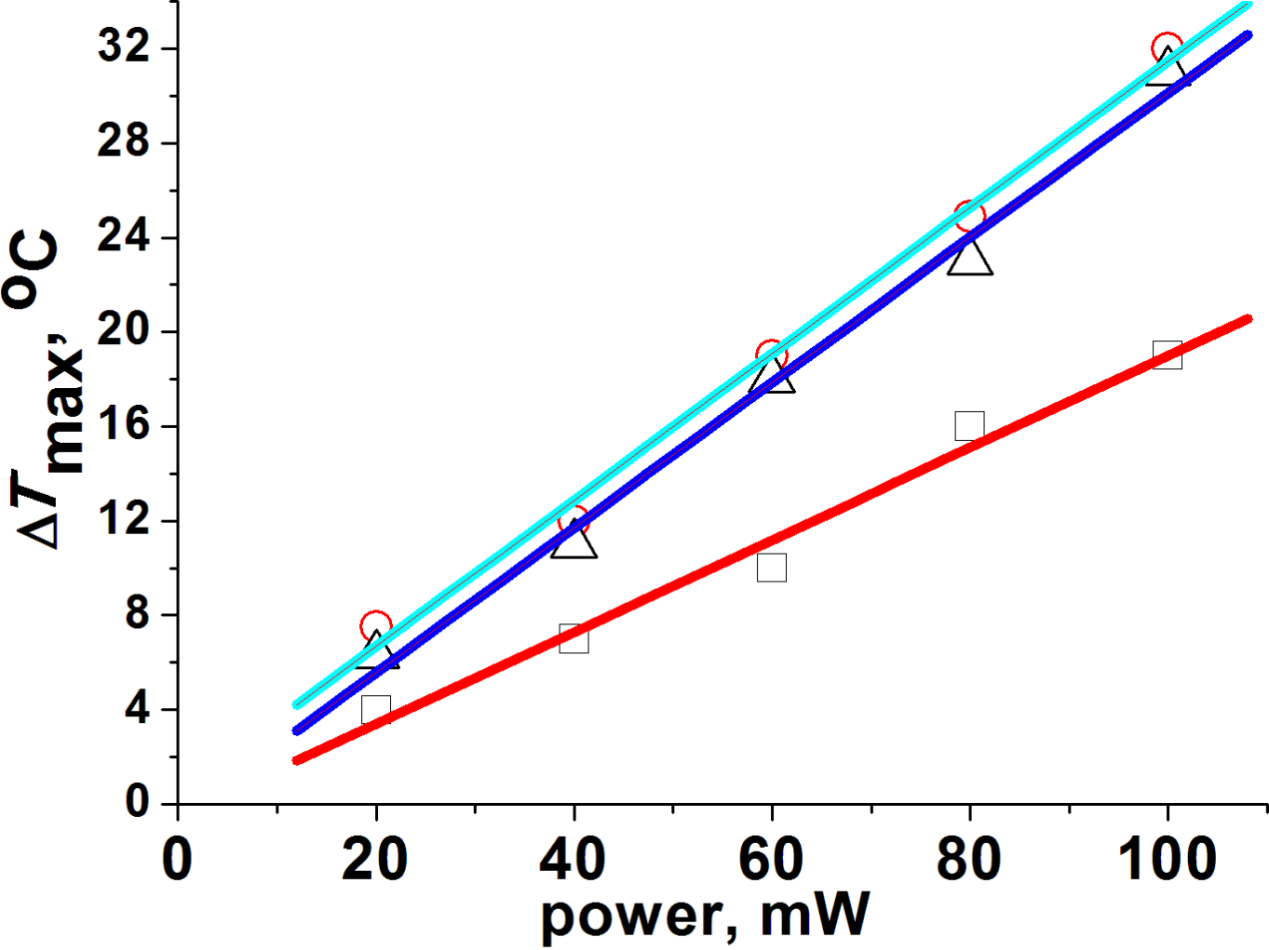
The photothermal effect (Δ*T* ± 1.5 °C) of PVA-GNS films upon NIR irradiation at 1064 nm (open squares), 800 nm (open circles) and 730 nm (open triangles). The data are fit with a linear function (colored solid lines).The slopes (*b*) of the fitting curves (*y* = *a* + *bx*) are: *b* = 0.19 ± 0.01 °C/mW (1064 nm); *b* = 0.3 ± 0.1 °C/mW (800 nm); *b* = 0.3 ± 0.1 °C/mW (730 nm). Irradiation area ≈ 0.4 cm^2^.

Having determined the mechanical properties of all fabricated films, we then investigated their photothermal properties. The NIR-laser-induced photothermal effect was studied by irradiating dry films exposed to air at room temperature at three different NIR wavelengths, namely λ = 730, 800 and 1064 nm. The chosen wavelengths fall around the maximum of the first LSPR peak (730 and 800 nm) and at the beginning (1064 nm) of the second LSPR peak of the corresponding films. As previously observed, in the case of GNS monolayers and printed surfaces, a steep temperature increase turning into a plateau after about 20 s was found also for PVA-GNS films (see example in Supporting Information File 1, Figure S3) [8,10]. As a control, no temperature increase was observed in blank PVA films without GNSs under NIR irradiation for powers up to 1 W at all the three wavelengths considered in this study (see an example in Supporting Information File 1, Figure S4). The resulting PVA films containing GNSs displayed a pronounced photothermal effect under irradiation at all three aforementioned laser wavelengths, as shown in Figure 4.

The large increase in temperature that is induced by the PVA-GNS films under NIR irradiation in comparison with aqueous GNS solutions [3] can be explained by the reduction of the thermal dissipation from the water (the conductivity of PVA is ≈0.31 ± 0.02 W/m∙K compared to the conductivity of water ≈0.6 W/m∙K), as previously reported [28]. Therefore, thin PVA films with no additional absorbance in the NIR range provide a favorable environment for localized increase of temperature.

**Figure 5:**
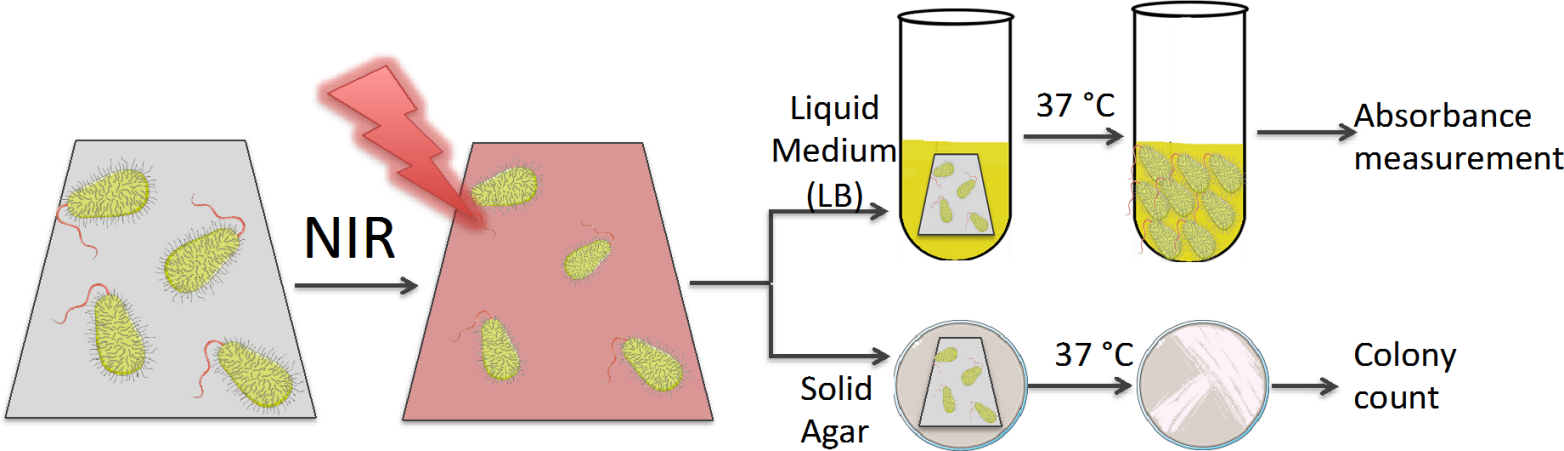
Workflow scheme of the antibacterial properties study.

In order to exploit the possibility that the photothermal properties of engineered PVA-GNS films may be used for antibacterial purposes, *Escherichia coli* (DH5-alpha strain) were inoculated onto PVA without GNSs (as control) and PVA-GNS films, and then exposed to NIR laser irradiation (using the second NIR window). The detailed experimental protocol of bacteria growth and NIR irradiation is provided in the Experimental section. The workflow of these experiments is schematically shown in Figure 5.

**Figure 6:**
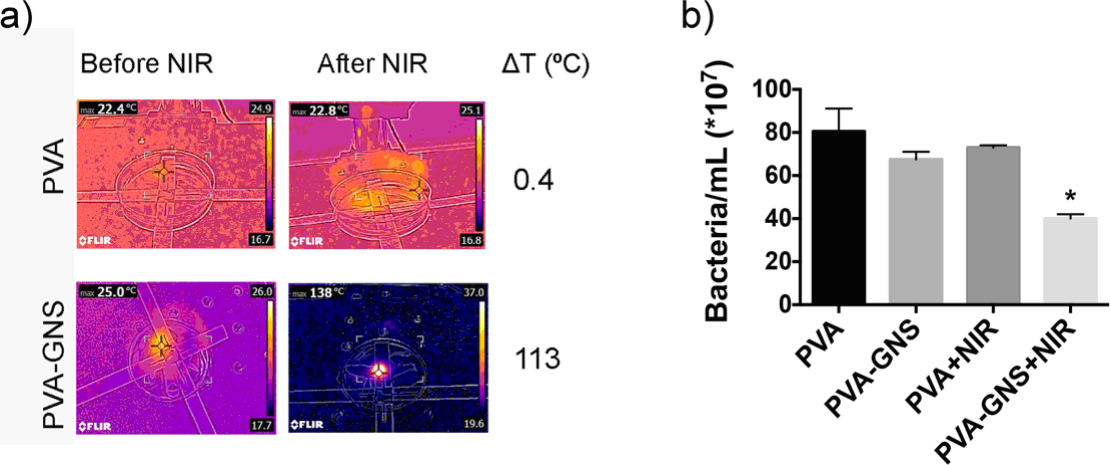
(a) Temperature increase of PVA and PVA-GNS films upon irradiation with a 1064 nm laser, as registered by a thermocamera (laser intensity 4 W/cm^2^). (b) After 5 minutes of laser irradiation (or simply exposure to the air for the control samples) the films were transferred to LB broth and left at 37 °C for cell growth. The number of bacteria per mL was calculated by spectrophotometric measurements at 600 nm (mean + SD). T-test against non-irradiated samples was performed (*p < 0.05).

In these first trials, the films were irradiated at 1064 nm. As the laser wavelength did not match the maximum absorbance of the GNSs, a higher laser intensity compared to the above-described experiments was chosen (laser power 1.3 W, intensity 4 W/cm^2^) to obtain an efficient increase in temperature. The membranes were irradiated for 5 min. These irradiation conditions produced a high local increase in temperature, as shown in Figure 6a. It can also be seen that blank PVA films did not show any temperature increase under the same irradiation conditions. Once irradiated, the inoculated membranes were transferred to solid or liquid Luria–Bertani (LB) by sterile tweezers and incubated at 37 °C and the growth of the inoculated medium was monitored over time by spectrophotometric measurements at 600 nm. Figure 6b shows similar bacteria growth from non-irradiated PVA or PVA-GNS films, meaning that the presence of GNS itself does not impact cell viability. By contrast, strong differences in the bacteria growth were observed in the case of PVA-GNS irradiated films. Under this condition, the growth decreased by approximately 50% (*p* < 0.05). When the films were cultured on solid LB media instead of LB broth (see Figure 5), similar antibacterial effects were observed. In this case, almost no colony growth could be observed on irradiated films containing GNSs, while a confluent growth was observed in all other conditions. Due to this confluent growth it was not possible to accurately quantify the antibacterial effect (see Figure S5, Supporting Information File 1). Even though gold is not intrinsically antibacterial, the photothermally induced local temperature increase was enough to inhibit bacterial growth viability and proliferation. Further studies will be performed whereby both the laser intensity and irradiation period are tuned, leading to improved antibacterial efficiency of the prepared films.

## Conclusion

In the present study PVA films containing PEGylated gold nanostars were fabricated. It was shown that the incorporation of PEGylated GNSs improved the mechanical properties of PVA films. The resulting films displayed a pronounced localized photothermal effect during NIR irradiation. It was also demonstrated that a laser-induced increase in temperature was efficient to eradicate bacteria grown on film surfaces. Thus, further studies will be focused on a detailed characterization of the dependence of the antibacterial efficacy on the duration, power and modality (continuous versus pulsed) of the irradiation. Great attention will be given to the optimization of GNS loading into the films, aiming to increasing the efficiency in bacteria and biofilm eradication under low laser power. In addition, photothermal-triggered controlled release of antibacterial compounds as a synergic effect will be also investigated. These strategies will lead to new, easily prepared, NIR-activated, reusable, antibacterial materials suitable for surface coating.

## Experimental

### Materials

PVA with an average molecular weight of 72000 g/mol (degree of hydrolysis 98%) was purchased from Sigma-Aldrich. Anhydrous citric acid and poly(ethylene glycol) (PEG-200) were commercially available from Fluka. Poly(ethylene glycol) thiols (<M_W_> = 5000 g/mol; SH-PEG_5000_–OCH_3_ and SH-PEG_5000_–COOH), polyethylene glycol *tert*-octylphenyl ether (Triton X-100), chloroauric acid, ascorbic acid, silver nitrate, and sodium borohydride were purchased from Sigma-Aldrich and used as received.

### Gold nanostar synthesis

As follows from [24,29], all glassware used for synthesis and further coating was pretreated with aqua regia before use. 5 mL of HAuCl_4_ 5 × 10^−4^ M in water were added to 5 mL of an aqueous solution of TritonX-100. Then, 0.6 mL of a previously ice-cooled solution of NaBH_4_ (0.01 M) in water were added. The mixture was gently hand-shaken and a reddish-brown color appeared. The seed solution was kept on ice and used within a few hours.

The growth solution was prepared in a 20 mL vial; 250 µL of AgNO_3_ (0.004 M) in water, 5 mL of HAuCl_4_ (0.001 M) in water were added in this order to 5 mL of an aqueous solution of Triton X-100 (0.2 M). Then, a 140–400 µL of an aqueous solution of ascorbic acid (0.0788 M) were added. The solution, after gentle mixing, became colorless. Soon after, 12 µL of the seed solution were added. The samples were allowed to equilibrate for 1 h at room temperature.

### PEGylation of gold nanostars

As described in [10], PEGylation of GNSs was carried out by simultaneously adding of 200 μL of a 10^−3^ M of aqueous solution of SH-PEG–OCH_3_ (<M_W_> = 5000 g/mol) to 10 mL of a GNS solution prepared as described above. The obtained solution was allowed to equilibrate for 3 h at room temperature while being gently shaken on a reciprocating shaker. The excess of PEG was removed by ultracentrifugation (25 min, 13000 rpm), the supernatant discarded, and the pellet redissolved in 10 mL of double-distilled water. The cleaning cycle was repeated twice to assure complete elimination of unbound PEG-SH and surfactant. After the ultracentrifugation step, GNSs were redissolved in 1 mL of Milli-Q water to increase the concentration of nanoparticles.

The coating with SH-PEG–COOH (<M_W_> = 5000 g/mol) was performed in the same way and the pH of the final solution was adjusted to ≈8 with 0.05 M NaOH.

### Preparation of PVA films containing gold nanostars

The films were prepared according to the following protocol: an aqueous solution of PVA (9% w/w) was stored one hour in an oven (≈100 °C) until complete dissolution of PVA, and then PEG-200 (0.11 g) was added and the mixture was stirred at ≈40 °C. 1.45 mL of PEGylated GNS solution were added dropwise and the mixture was stirred 5 h at ≈40 °C. Then, citric acid (0.1 g) was added and 5 mL of the resulting solution were cast into a Petri dish. Once the film was formed, it was sintered 20 min at 130 °C to complete crosslinking. The samples were dried at room temperature for four days and kept in a desiccator before further measurements.

### Measurement of film thickness

The thickness of the films was measured using a hand-held micrometer (EK-1619; EKTools). Five replications were conducted for each sample. Seven measurements were taken in different positions around the sample and the average value was calculated.

### UV/VIS/NIR spectroscopy and film transparency

The extinction spectra of GNS solutions and GNS-containing films were recorded using a UV/VIS/NIR spectrophotometer V-570 (Jasco).

### Microscopic images of PVA-GNS films

Raster x–y-images were acquired with a Leica SP5 TCS confocal microscope (Leica Microsystems, Wetzlar, Germany). Each image was acquired through a 40× oil objective at 400 Hz, with1024 × 1024 pixels, line average 4, in reflection mode with λ_exc_ = 488 nm, NA = 1.3.

By acquiring a z-stack of images it is possible to estimate the total number of particles in a known volume, and therefore the density of particles in the film. A z-stack was acquired at zoom 15, covering a volume of view of 25.8 × 25.8 μm, 1 μm. The number of spots in each plane can be estimated by employing the Fiji plugin TrackMate (ImageJ, open source image processing software). By taking into account 20 consecutive planes, the total number of spots contained in a volume *V* = 25.8 × 25.8 × 1 μm × 20 = 13313 μm^3^ is obtained. A correction was applied since each spot can be counted for four consecutive planes due to the extension of the point spread function of the microscope in reflection mode along the optical axis.

We found that the number of spots identified was randomly dispersed around the average value irrespective of the distance from the film surface within 15%. Since the optical resolution is about 300 nm, we cannot ascertain that the detected spots in the confocal image were single nanoparticles. The distribution of the reflectivity of the spots on each layer did not change more than a few tens of percent units, indicating that if any aggregation is occurring it is very monodisperse. It is hardly conceivable that all of the observed spots correspond to the same aggregation number, unless these are single nanoparticles.

### Swelling behavior

As described previously [20], in order to estimate the swelling degree of the films, three specimens of each film (2 × 2 cm) weighed before (*W*_0_) were separately soaked in 7 mL of Milli-Q water for 24 h. Then, the water was removed and the samples were gently wiped with filter paper. After this, the weight of the swollen samples was determined (*W*_1_). The water content (*W*_Q_) was determined as the average value of each measurement according to following equation:

[1]
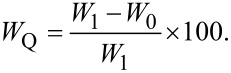


The swelling degree (*S*_w_) was evaluated using following equation:

[2]
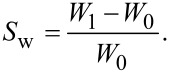


### Weight loss

Three specimens of each film (20 × 20 mm^2^) weighed before (*W*_0_) were separately soaked in 20 mL of Milli-Q water for four days at room temperature. Then each sample was re-dried and weighed again (*W*_1_). The resulting weight loss (*W*_L_) was estimated using following equation:

[3]
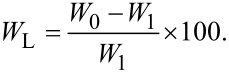


### Mechanical properties

Dried specimens (*n* = 3 per type of tested films) were cut into a rectangular shape (20 × 5 mm^2^). The mechanical properties were evaluated by uniaxial tensile tests using a dynamic mechanical analyzer (DMA, Q800, TA Instrument) equipped with a tensile clamp. Tests were performed at 37 °C by applying a ramp force of 1 N/min up to 18 N (force limit of DMA instrument).

The elastic modulus (*E*), calculated considering the strain range 0–10%, maximum stress (σ_max_) and maximum strain (ε_max_) were drawn from the stress–strain curve elaboration and expressed as mean ± standard deviation of the values of the three specimens tested.

### Statistical analysis

Statistical analysis (GraphPad Prism 6.0 software) was performed using a t-test (Student’s test), with significance level *p* = 0.05. Normal distribution was verified by normal probability plots.

### Photothermal effect upon NIR irradiation

The films were irradiated with NIR light at λ = 800 nm (Tsunami, Spectra Physics, CA, USA; pulse repetition rate 80 MHz, pulse width 200 fs), at λ = 730 nm (Mai Tai, Spectra Physics, USA) and at λ = 1064 nm (temperature-controlled laser diodes, ThorLabs, Germany or 1064 Ventus, Laser Quantum, UK). The substrate temperature change was monitored by means of a thermocamera (FLIR, E40, USA) and the supporting analysis software. The temperature was monitored with respect to time on a region of interest (ROI) comprising the irradiated area and the maximum temperature within the ROI was taken as a measure of the temperature increase. The photothermal effect was measured at four different points of each film. The emissivity of the films is 1.3% higher than water (for which the thermocamera was calibrated), justifying the reliability of remote measurement by the thermocamera [9–10].

### Testing of antibacterial effect upon NIR irradiation

As a Gram-negative model bacteria, *Escherichia coli DH5-alpha* were selected. A single bacterial colony was grown in LB broth medium under shaking (180 rpm) at 37 °C for 15 h. The bacteria concentration was calculated by spectrophotometric measurement (SmartSpec Plus, Biorad) at 600 nm, considering that 8 × 10^8^ cells/mL have an optical density of 1 at 600 nm. A dilution in LB broth from this culture was used for the following tests, corresponding to a concentration of 1 × 10^5^ cells/mL.

Blank PVA or PVA-GNS films were precut to fit a 48-well microplate well. 20 μL of diluted bacterial suspension was inoculated into the wells containing either blank PVA or PVA-GNS films. The films were dried under a hood and irradiated (or not) for 5 minutes using a 3 W Ventus 1064 laser, Laser Quantum (UK) operating at a power of 1300 mW, which illuminates the sample with a power per unit of area of ≈4 W/cm^2^ at the sample position. Sterile tweezers were used to transfer the membranes to LB agar plates or to LB broth and left to grow at 37 °C. The LB antibacterial effect was estimated on images acquired on LB agar plates, while the bacteria concentration from LB broth was calculated by spectrophotometric measurements as explained before. At least two biological replicas were conducted to calculate the mean and standard deviation. Statistical analysis was performed using GraphPad Prism v.6.

## Supporting Information

File 1 Additional data.
