# Supplementary material for: Fabrication of photothermally active poly(vinyl alcohol) films with gold nanostars for antibacterial applications
Source: Beilstein J Nanotechnol. 2018 Jul 23;9:2040–8. doi: 10.3762/bjnano.9.193 (PMC6071708; doi:10.3762/bjnano.9.193)
Supplement: File 1 — Additional data. [file Beilstein_J_Nanotechnol-09-2040-s001.pdf]

Supporting Information

for

Fabrication of photothermally active poly(vinyl alcohol) films with gold nanostars for antibacterial applications

Mykola Borzenkov*[1], Maria Moros*[2], Claudia Tortiglione[2], Serena Bertoldi[3,4], Nicola Contessi[3,4], Silvia Faré[3,4], Angelo Taglietti[5], Agnese D'Agostino[5], Piersandro Pallavicini[5], Maddalena Collini[1,6], and Giuseppe Chirico[1,6]

Address: [1]Department of Medicine and Surgery, Nanomedicine Center, University of Milano-Bicocca, Piazza dell'Ateneo Nuovo, 20126, Milan, Italy, [2]Institute of Applied Sciences and Intelligent Systems, National Research Council of Italy, Via Campi-Flegrei 34, 80078, Pozzuoli, Italy, [3]Politecnico di Milano, Department of Chemistry, Materials and Chemical Engineering "Giulio Natta", Piazza Leonardo da Vinci 32, 20133, Milan, Italy, [4]Local Unit Politecnico di Milano, INSTM, Consorzio Nazionale di Scienza e Tecnologia dei Materiali, Milan, Italy, [5]Department of Chemistry and Centre for Health Technology, University of Pavia, Viale Taramelli 12, 27100, Pavia, Italy, and [6]Department of Physics "G. Occhialini", University of Milano-Bicocca, Piazza dell'AteneoNuovo, 20126, Milan, Italy

Email: Mykola Borzenkov* - mykola.borzenkov@unimib.it; Maria Moros* - m.moros@isasi.cnr.it

Addional Data

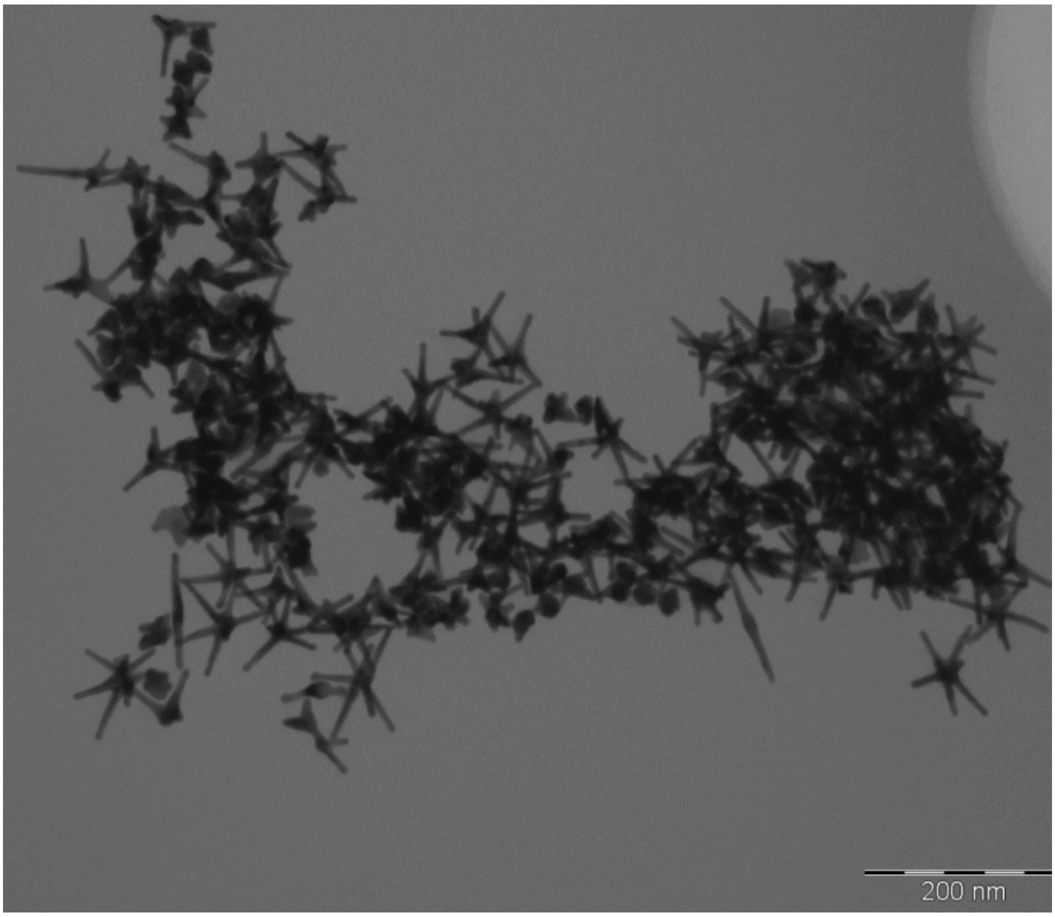

Figure S1: Example of TEM image of gold nanostars synthesized in the presence of non-ionic surfactant Triton X-100.

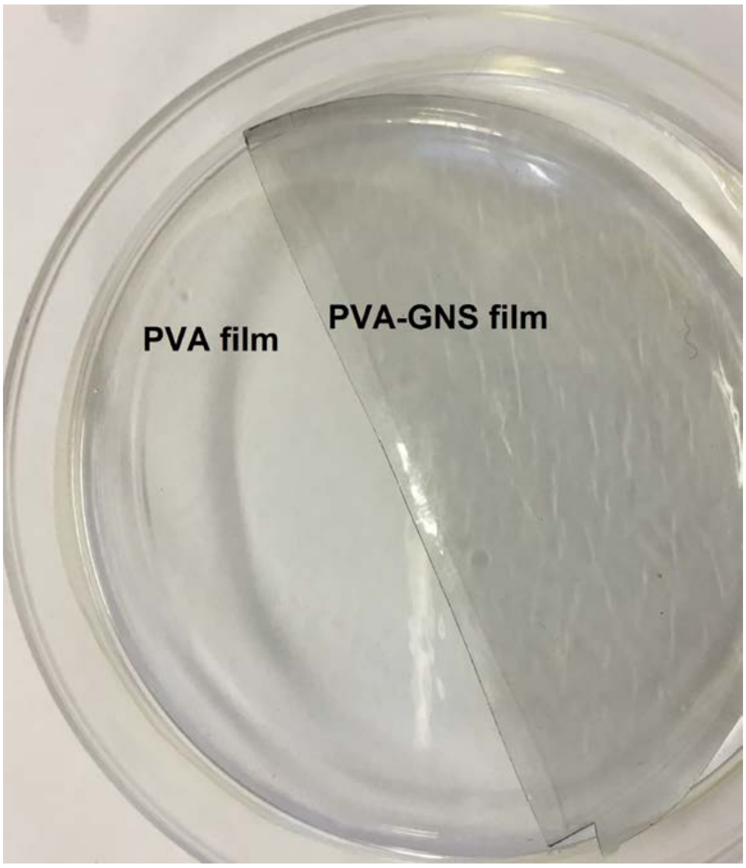

Figure S2: Photo of fabricated blank PVA and PVA-GNS films.

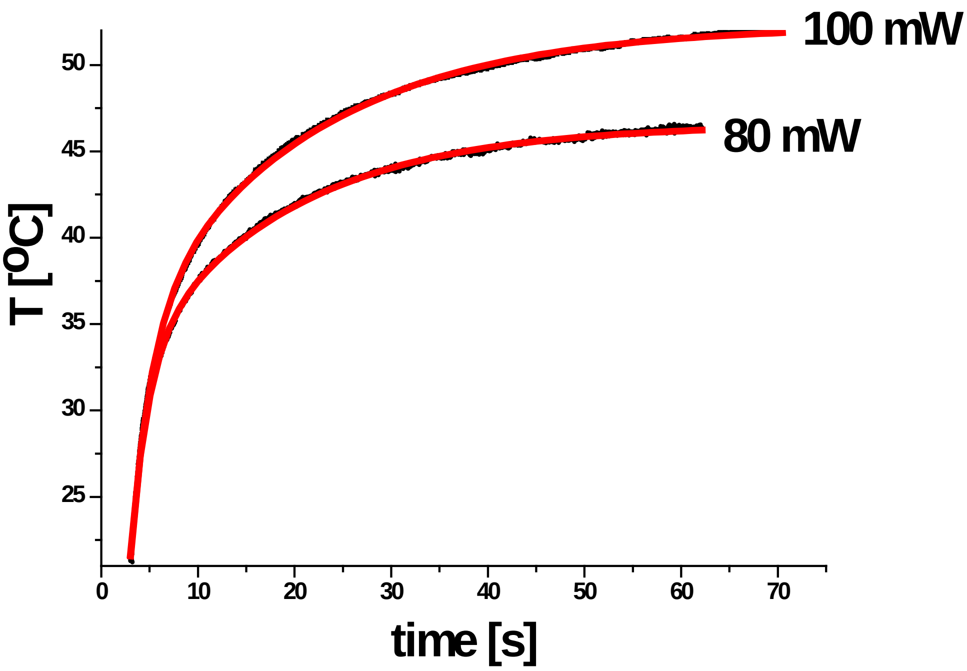

Figure S3: The increase of temperature of PVA-GNS film upon irradiation with 800 nm laser (laser powers are 80 mW and 100 mW). The red line is the double exponential best fit function to the data.

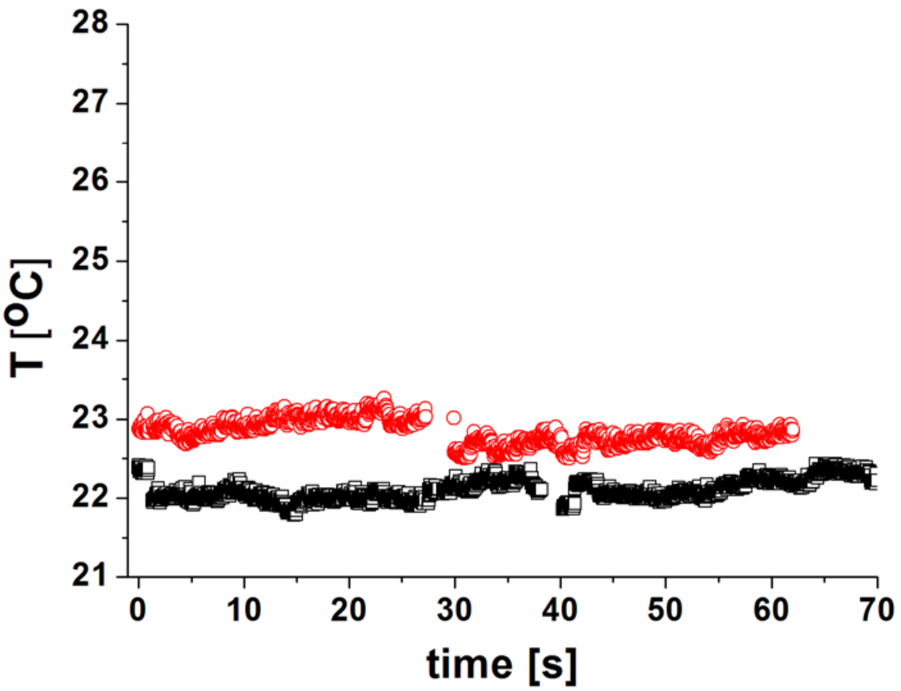

Figure S4: The changes of temperature of blank PVA film upon irradiation with 800 nm laser. Laser powers are 100 mW (open squares) and 400 mW (open circles). The mismatch in the time scale at 38 s (open squares) and 28 s (open circles) correspond to the auto-calibration procedure of the thermocamera.

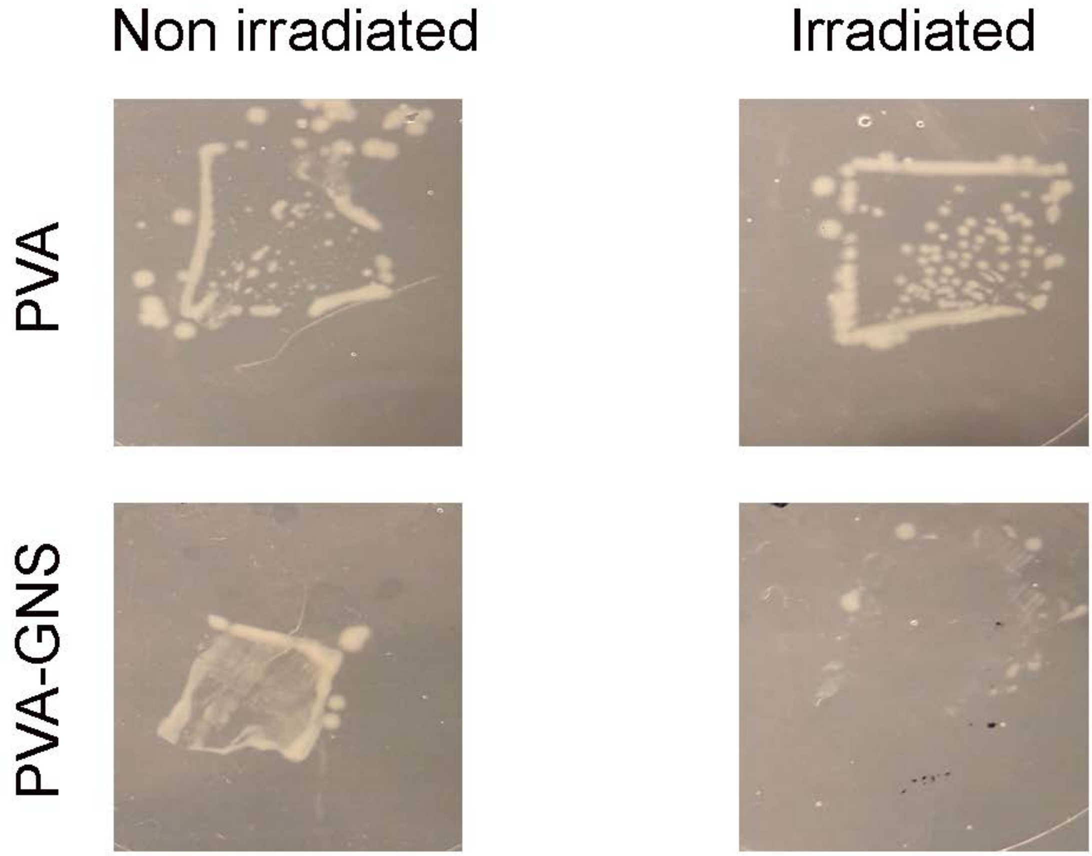

Figure S5: Blank PVA and PVA-GNS films inoculated with *E.coli*, either irradiated or not were transferred to LB agar plates and allowed to growth at 37 ºC. Bacteria growth can be observed as single or confluent colonies, mirroring the film edge shape. A clear antibacterial effect was induced in irradiated PVA-GNS, as shown by the almost total absence of bacterial growth, although a precise quantification was not possible due to bacterial confluence growth.
